# Public health round-up

**DOI:** 10.2471/BLT.19.010419

**Published:** 2019-04-01

**Transforming WHO**

World Health Organization Director-General Tedros Adhanom Ghebreyesus presenting the plan to reform WHO at a 6 March all-staff meeting.

**Figure:**
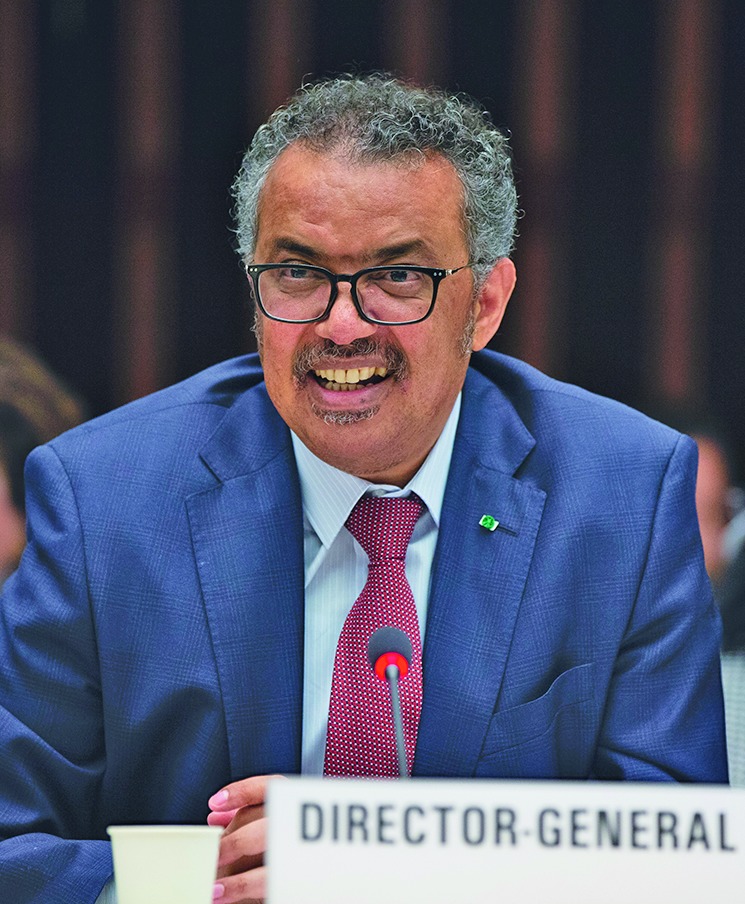
WHO/Christopher Black

## WHO announces major reform

World Health Organization (WHO) Director-General Tedros Adhanom Ghebreyesus announced a major reform of WHO’s structure and operating model on 6 March. The main aim of the reform is to align headquarters, regional offices and country offices’ efforts to achieve WHO’s “triple billion” targets set out in the 13th General Programme of Work 2019-2023.

Changes include the creation of a new Division of the Chief Scientist and improved career opportunities for WHO scientists.

A new Department of Digital Health will support countries in their efforts to harness the power of digital health and innovation, supporting efforts to assess, integrate, and regulate digital technologies and artificial intelligence.

A new WHO Academy will be a part of plans to develop a dynamic and diverse health workforce. The academy will provide new learning opportunities for staff and public health professionals globally.

WHO’s work to support countries in preventing and mitigating the impact of outbreaks and other health crises will be strengthened by creating a new Division of Emergency Preparedness, as a complement to WHO’s existing work on emergency response.

A corporate approach to resource mobilization will be reinforced, aligned with strategic objectives and driving new fundraising initiatives to diversify WHO’s funding base, reduce its reliance on a small number of large donors and strengthen its long-term financial stability.

“Our vision remains the same as it was when we were founded in 1948: the highest attainable standard of health for all people. But the world has changed, which is why we have articulated a new mission statement for what the world needs us to do now: to promote health, keep the world safe and serve the vulnerable,” the Director-General said.

The Director-General also announced the appointment of Dr Zsuzsanna Jakab as WHO Deputy Director-General. 

https://www.who.int/news-room/detail/06-03-2019-who-unveils-sweeping-reforms-in-drive-towards-triple-billion-targets

## WHO reaffirms commitment to Ebola response

WHO Director-General Tedros Adhanom Ghebreyesus travelled to the Democratic Republic of the Congo in March to see first-hand the efforts being made by WHO teams and partners to contain the Ebola outbreak in the north-east of the country. 

The visit came in the wake of two attacks on Médecins Sans Frontières-run Ebola treatment centres, which took place on 24 and 27 February in the towns of Katwa and Butembo.

“We are committed to ending the outbreak, and we are committed to improving the health of the people of the Democratic Republic of the Congo,” Dr Tedros said during a 9 March visit to the Butembo Ebola treatment centre.

The attacks occurred in areas with ongoing transmission of Ebola in the community and could set back efforts to contain transmission. The violence and disruption to the treatment centres also make it difficult for Ebola responders to carry out their work.

WHO is working closely with its partners to determine appropriate action to ensure the overall Ebola response is maintained. On 26 February Dr Tedros called on donors to ensure that the response to the Ebola outbreak had the funding needed to fully implement the Strategic Response Plan interventions, which cover a 6-month period and have a budget of US$ 148 million. As of 5 March, only US$ 30 million had been received.

https://www.who.int/news-room/detail/09-03-2019-who-director-general-reiterates-commitment-to-ebola-response-despite-another-attack

https://www.who.int/csr/don/7-march-2019-ebola-drc/en/

## New global influenza strategy

Last month WHO released a *Global influenza strategy* for 2019-2030 aimed at protecting people in all countries from the threat of influenza. The goal is to prevent seasonal influenza, control the spread of influenza from animals to humans, and prepare for the next influenza pandemic.

Released on 11 March, the strategy encourages countries to build tailored influenza programmes that contribute to national and global preparedness and calls for increased capacity for disease surveillance and response, prevention and control. It also calls for better tools to prevent, detect, control and treat influenza, such as more effective vaccines and antivirals.

Influenza remains one of the world’s greatest public health challenges. Every year across the globe, there are an estimated 1 billion cases, of which 3 to 5 million are severe cases, resulting in 290 000 to 650 000 influenza-related respiratory deaths. WHO recommends annual influenza vaccination as the most effective way to prevent influenza. Vaccination is especially important for people at higher risk of serious complications and for health care workers.

https://www.who.int/news-room/detail/11-03-2019-who-launches-new-global-influenza-strategy

## Zoonotic diseases guide launched

The Food and Agriculture Organization of the UN (FAO), the World Organisation for Animal Health (OIE), and WHO launched a guide entitled *Taking a multisectoral, one health approach: a tripartite guide to addressing zoonotic diseases in countries* on 11 March.

Every year, nearly 60 000 people die from rabies. Other zoonotic diseases such as avian influenza, Ebola and Rift Valley fever constitute additional threats. 

These diseases not only affect human health, but also animal health and welfare, causing lowered productivity or death, and consequently affecting farmers’ livelihoods and national economies. 

To face these challenges a One Health approach is needed.

http://www.oie.int/fileadmin/Home/eng/Media_Center/docs/EN_TripartiteZoonosesGuide_web.pdf

## Polio emergency extended

WHO extended the international spread of poliovirus as a Public Health Emergency of International Concern (PHEIC) by a further three months, following a recommendation by the International Health Regulations Emergency Committee. 

The committee made the recommendation on 1 March, citing the increasing number of people infected with wild poliovirus in Afghanistan and Pakistan (the only two countries where wild poliovirus transmission continues) as a matter of concern.

The committee also expressed concern regarding vaccine-derived polio outbreaks. Eight countries in four WHO Regions are currently responding to outbreaks of vaccine-derived polio, the highest number in recent years.

The committee reported that the number of countries in which immunization systems have been weakened or disrupted by conflict and complex emergencies poses a growing risk. Surveillance gaps were also cited as matters of concern, as was population movement. A regional approach supported by strong cross-border cooperation is required to respond to these risks.

https://www.who.int/news-room/detail/01-03-2019-statement-of-the-twentieth-ihr-emergency-committee

**Cover photo**

A resident of a village near Surikovo in the Krasnoyarsk district of the Russian Federation waits to see doctors staffing the mobile clinical diagnostic centre on the St Luka health train.

**Figure:**
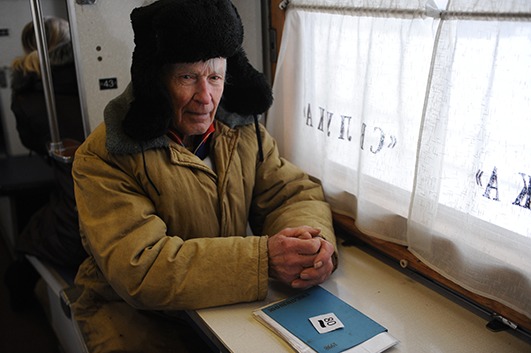
WHO/Victoria Ivleva

## Boost for emergency fund

Japan provided US$ 22 million to WHO’s Contingency Fund for Emergencies (CFE), the single largest contribution to the fund since its founding in 2015.

Contributions to the CFE are not earmarked for specific initiatives, giving WHO the flexibility required to act quickly in response to disease outbreaks, natural disasters and humanitarian emergencies. A quick response not only saves lives and helps prevent unnecessary suffering, it also dramatically reduces the costs of controlling outbreaks and emergencies and reduces wider social and economic impacts.

In 2018 the CFE was the source of US$ 37.5 million used to respond to 28 health emergencies, with most allocations released within 24 hours. For example, CFE support helped WHO immediately send teams to respond to two Ebola outbreaks in the Democratic Republic of the Congo; assist government efforts to stop a Lassa fever outbreak in Nigeria; and provide support for the earthquake response in Papua New Guinea.

Ensuring the fund’s sustainability strengthens global health security. WHO is working with Member States to reach the CFE’s target of US$ 100 million over the 2018-2019 biennium.

https://www.who.int/about/planning-finance-and-accountability/financing-campaign/japan-strengthens-global-health-security

## WHO launches hearing app

WHO launched "hearWHO" 1 March, a free software application (app) for mobile devices targeted at those who are at risk of hearing loss or who already experience related symptoms. Most acquired hearing loss is caused by occupational exposure to loud noise, meningitis, ototoxic medications, infections, and age-related cochlear degeneration.

Users of the app are asked to listen to recordings of numbers which have been recorded against varying levels of background sound, simulating listening conditions in everyday life. They then enter the numbers into their mobile devices when prompted. 

The app displays the users' score and its meaning and stores the outcome of the test so that the user can monitor hearing status over time. The app can be used by individuals as well as health providers with a view to facilitating hearing screening especially in low-resources settings.

Early detection of hearing loss is crucial to identifying the most appropriate responses. These include prevention measures such as loud noise avoidance, supportive interventions, including captioning and sign language, and assistive interventions such as hearing aids and cochlear implants. 

https://www.who.int/deafness/news/hearWHOApp-news/en/

**Looking ahead**

9 – 10 April – WHO European High-level Conference on Noncommunicable Diseases Ashgabat, Turkmenistan

28 Apr – 1 May – 26th European Congress on Obesity 2019, Glasgow, Scotland

20 – 28 May – World Health Assembly, Geneva, Switzerland

3 – 6 June – Women Deliver 2019 Global Conference, Vancouver, Canada

11 – 13 June – High-level meeting on health equity, Ljubljana, Slovenia

